# BNIP3 acts as transcriptional repressor of death receptor-5 expression and prevents TRAIL-induced cell death in gliomas

**DOI:** 10.1038/cddis.2013.100

**Published:** 2013-04-11

**Authors:** T R Burton, E S Henson, M B Azad, M Brown, D D Eisenstat, S B Gibson

**Affiliations:** 1 Departments of Biochemistry and Medical Genetics, University of Manitoba, Winnipeg, Manitoba, Canada; 2 Manitoba Institute of Cell Biology, University of Manitoba, Winnipeg, Manitoba, Canada; 3 Departments of Pediatrics and Child Health, University of Manitoba, Winnipeg, Manitoba, Canada; 4 Departments of Human Anatomy and Cell Science, University of Manitoba, Winnipeg, Manitoba, Canada

**Keywords:** BNIP3, TRAIL, gliomas, DR5, apoptosis

## Abstract

Glioblastoma multiforme (GBM) is the most common and malignant brain tumor, and current treatment modalities such as surgical resection, adjuvant radiotherapy and temozolomide (TMZ) chemotherapy are ineffective. Tumor necrosis factor-related apoptosis-inducing ligand (TRAIL) is a novel cancer therapeutic agent for GBM because of its capability of inducing apoptosis in glioma cells. Unfortunately, the majority of glioma cells are resistant to TRAIL-induced apoptosis. The Bcl-2 nineteen kilodalton interacting protein (BNIP3) is a pro-cell death BH3-only member of the Bcl-2 family that is one of the highest expressed genes in hypoxic regions of GBM tumors. We previously found that BNIP3 is localized to the nucleus in GBM tumors and suppresses cell death in glioma cells. Herein, we have discovered when BNIP3 nuclear expression is knockdown in glioma cell lines and in normal mouse astrocytes, TRAIL and its death receptor, death receptor-5 (DR5) expression is increased. In addition, when nuclear BNIP3 expression is increased, the amount of TRAIL-induced apoptosis is reduced. Using a streptavidin pull-down assay, we found that BNIP3 binds to the DR5 promoter and nuclear BNIP3 binds to the DR5 promoter. Furthermore, nuclear BNIP3 expression in GBM tumors correlates with decreased DR5 expression. Taken together, we have discovered a novel transcriptional repression function for BNIP3 conferring a TRAIL resistance in glioma cells.

Glioblastoma multiforme (GBM) is the most malignant form of brain cancer with a high mortality rate.^1^ The median duration of survival for patients with GBM is less than 15 months even with treatment that usually consists of a combination of surgery, radiation and chemotherapy such as temozolomide (TMZ).^2^ GBMs are considered members of the group of diffuse infiltrating gliomas.^3^ Of this group, GBMs are the most common representing over 40% of adult gliomas. The pathological characteristics of GBM tumors are nuclear atypia, cellular polymorphism, significant mitotic activity, microvascular proliferation and extensive regions of necrosis that indicate areas of hypoxia.^1, 3^

Tumor necrosis-related apoptosis-inducing ligand (TRAIL/Apo-2 L) binds to death receptor-4 (DR4, TRAIL-R1) and death receptor-5 (DR5, TRAIL-R2). This lead to the activation of caspase 8 and subsequent cleavage of the Bcl-2 BH3-only family member BID causing mitochondrial dysfunction and apoptosis. TRAIL induces apoptosis in cancer cells without inducing apoptosis in normal non-transformed cells. This provides the potential for TRAIL to become a therapeutic agent in the treatment of cancer.^4, 5^ Levels of DR4 and DR5 expression are thought to contribute at least in part to the amount of TRAIL-induced apoptosis.^5, 6, 7, 8^ In GBM tumors, the expression levels of DR4 and DR5 are correlated with increased survival of patients, suggesting that increased DR4 and DR5 contributes to TRAIL-induced cell death in GBM tumors.^9^ Unfortunately, many glioma cells are TRAIL resistant.^10, 11^ Hence, understanding the regulation of TRAIL receptor activation could provide insight into increasing TRAIL-induced apoptosis in these TRAIL-resistant cells.

Many chemotherapeutic agents increase TRAIL death receptor expression contributing to their cytotoxicity in cancer cells.^5, 12, 13^ In addition, this increase in death receptors causes a synergistic apoptotic response when combined with TRAIL.^13, 14, 15, 16^ We and others have determined that upregulation of DR4 and DR5 following treatment with DNA damaging agents and histone deacetylase (HDAC) inhibitors (histone deacetylase inhibitors) is mediated by transcription factors NF-*κ*B and p53, contributing to increased apoptosis.^6, 17, 18, 19^ However, the regulation of DR5 expression levels and its impact on cell death is less well understood and needs further studies.

The Bcl-2 nineteen kilodalton interacting protein 3 (BNIP3) is a pro-cell death Bcl-2 family that is upregulated during hypoxia.^20^ When BNIP3 is upregulated, it induces caspase-independent cell death by localizing to the mitochondria and opening the permeability transition pore leading to loss of mitochondrial membrane potential (Δψm) and reactive oxygen species production.^20, 21^ Recently, studies have implicated BNIP3 in the induction of autophagic cell death (programmed cell death type II) in malignant gliomas in response to hypoxia and arsenic trioxide treatment.^22, 23^ BNIP3 is directly upregulated under hypoxic conditions by the transcription factor HIF-1, contributing to hypoxia-induced cell death.^24, 25, 26^ Paradoxically, BNIP3 is expressed at high levels in viable cells within hypoxic regions of tumors.^27^ This is partially due to nuclear localization of BNIP3 in tumors where BNIP3 fails to associate with the mitochondria, and promote cell death.^28^ This nuclear localization allows BNIP3 to associate with various promoters and repress their expression. We have found that BNIP3 binds to and represses the expression of apoptosis-inducing factor (AIF-1), contributing to resistance against TMZ-induced apoptosis.^29^

Herein, we have discovered that BNIP3 localized to the nucleus represses DR5 expression and blocks TRAIL-induced apoptosis in glioma cells. In GBM tumors, nuclear BNIP3 expression correlates with lower levels of DR5 expression. This provides evidence for a novel mechanism for TRAIL resistance in glioma cells.

## Results

### Nuclear BNIP3 represses DR5 expression

**Figure 1:**
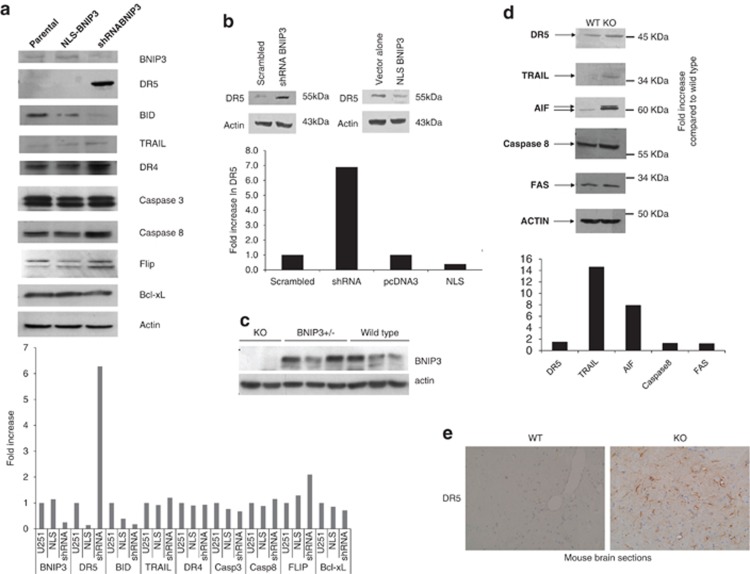
TRAIL signaling proteins are altered when BNIP3 is upregulated in the nucleus or knocked down. (**a**) Total cell lysates from U251 parental, U251 overexpressing BNIP3 targeted to the nucleus (BNIP3-NLS) and BNIP3 knocked down (BNIP3-shRNA) were western blotted for BNIP3, DR5, BID, TRAIL, DR4, caspase 3, caspase 8, FLIP, Bcl-xL and actin as a loading control. These blots represent three independent experiments. (**b**) U87 cells were transfected with BNIP3-NLS expression vector, vector alone, shRNA against BNIP3 or scrambled vector. Lysate was western blotted for DR5 and actin as loading control. (**c**) Protein was isolated from mature astrocytes as described above and then western blotted for DR5, TRAIL, AIF and 8 and FAS. Blots were stripped and reprobed for actin as a loading control. (**d**) Immunohistochemistry was performed on 5 *μ*M sections from the brains of 30-week BNIP3 wild type and knockout mice using the DAKO EnVision system and DR5 antibody (Atlas). Images were taken in matched regions of the hippocampus in both animals. Scale bar indicates 100 *μ*M. (**e**) Total cell lysates were generated from cryopreserved brain tissue extracted from wild type, heterozygous and BNIP3-null adult mice. Lysates were analyzed for BNIP3 expression by western blot, with actin as a loading control; each lane represents a different mouse. Mice were killed by cervical dislocation to minimize hypoxia at the time of death, and brain tissue was removed within 5 min

We have previously identified that BNIP3 is expressed in the nucleus of primary human astrocytes and glioma cell lines, and it transcriptionally represses the expression of AIF.^28^ To further characterize genes transcriptionally repressed by BNIP3, we conducted Affymetrix oligonucleotide microarray analysis on total RNA from U251 and U251shRNABNIP3 cells on Affymetrix GeneChip Human U133 Plus 2.0 microarray chips. Raw GeneChip data were analyzed using the GeneSifter software (www.GeneSifter.net) to determine the fold change of gene expression between U251 parental and U251shRNABNIP3 cells (Supplementary Figure 1). We identified several genes upregulated in the TRAIL apoptotic pathway, including DR5. To confirm these results, we western blotted for protein in the TRAIL apoptotic pathway in U251 stably transfected cell lines, expressing different levels of BNIP3 (nuclear localization signal (NLS)-BNIP3 has higher levels of BNIP3 in the nucleus and short hairpin RNA (shRNA) BNIP3 has lower levels of total BNIP3). We found that DR5 expression significantly increased in cells lacking BNIP3 expression, whereas TRAIL, DR4, caspase 8, caspase 3, Bcl-xL and FLIP expression remained unchanged. BID expression was reduced in cell lacking BNIP3 (Figure 1a). As a control, we found the non-targeting BNIP3 failed to increased DR5 expression and decrease BNIP3 expression (Supplementary Figure 2). We also found similar result in increased DR5 expression in U87 cells overexpressing NLS-BNIP3 and knocked down for BNIP3 expression (Figure 1b). As a control, non-targeting siRNA also failed to reduce BNIP3 expression (Supplementary Figure 3). We further confirmed these results in mouse astrocytes isolated from knockout mice lacking BNIP3 expression. We found that in BNIP3 knockout cells lacked BNIP3 expression, whereas the heterozygote (BNIP3+/−) and wild-type (wt) mice brains expressed a significant amount of BNIP3 (Figure 1c).The expression levels of DR5 and TRAIL were increased, whereas there was little change in FAS receptor and caspase 8 in astrocytes lacking BNIP3 expression (KO) compared with wild type. Consistent with our previous study, AIF expression was increased in astrocytes lacking BNIP3 expression (Figure 1d). Finally, wild type or BNIP3 knockout mouse brain sections were fixed and immunostained with a DR5 antibody. In agreement with western blotting analysis, DR5 protein levels were increased in brain sections lacking BNIP3 expression (Figure 1e).

### Nuclear BNIP3 represses the expression of DR5 mRNA expression

**Figure 2:**
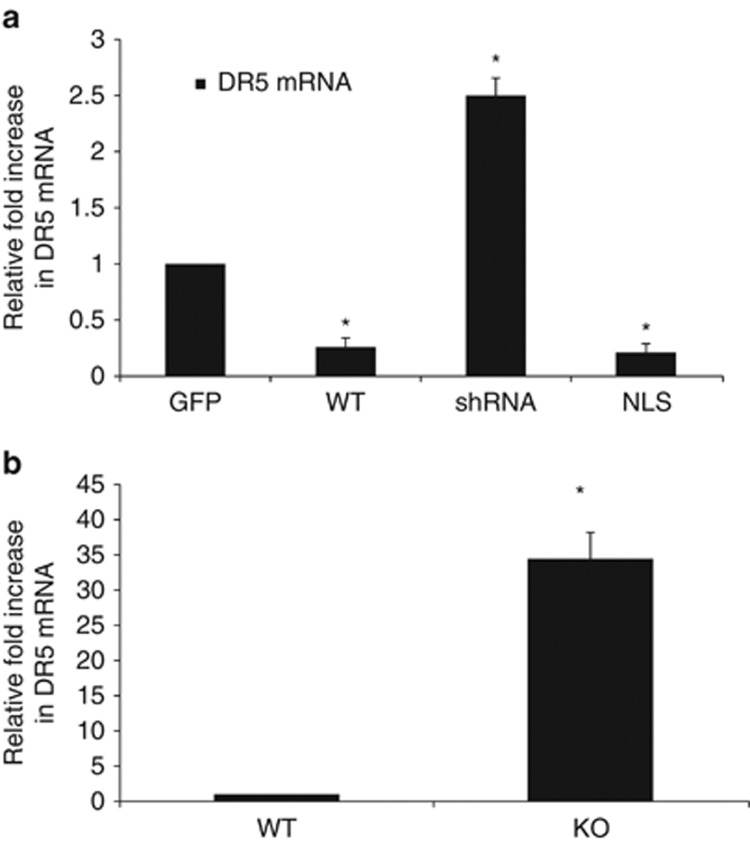
Nuclear BNIP3 suppresses the expression of DR5 at the RNA level. (**a**) U251 cells were transiently transfected with GFP (control), wild-type BNIP3, shRNA against BNIP3 and BNIP3 targeted to the nucleus (NLS). Fold changes in the expression of DR5 mRNA were determined using the ΔΔC_T_ method and all fold changes were normalized to the GFP transfection control. Human cyclophilin was used as the control gene for the experiment. Error bars represent the S.E. determined from three independent experiments. * denotes a *P*-value <0.05 representing statistical significance between wild-type BNIP3-transfected cells, shRNA and BNIP3-NLS-transfected cells compared with GFP-transfected cells. (**b**) Total RNA was isolated from the brains of BNIP3 wild-type or knockout mice. Fold changes in the expression of DR5 mRNA were determined using the ΔΔC_T_ method and all fold changes were normalized to the wild-type mouse. Mouse 18s was used as the control gene for the experiment. Error bars represent the S.E. determined from three independent experiments. * denotes a *P*-value <0.001 representing statistical significance between wild-type BNIP3 mice and BNIP3 knockout mice

As DR5 protein was increased when BNIP3 expression was knocked down and it is a target for cancer therapy,^10^ we then sought to determine whether DR5 mRNA levels are regulated by nuclear BNIP3. We measured DR5 mRNA levels in the U251-transfected cell lines as described above. We found that the NLS-BNIP3-expressing cells had reduced DR5 mRNA expression compare with cells transfected with GFP (control cells). Concurrently, shRNA–BNIP3-expressing cells had significantly increased DR5 mRNA levels (Figure 2a) compared with parental controls. When wild-type BNIP3 was overexpressed in the cells, we observed a decrease in DR5 mRNA expression compared with control cells (Figure 2a). To confirm these results, we also used total RNA from mouse brains and determined the level of DR5 expression. We found that DR5 mRNA expression was increased compared with wild-type brains. This suggests that BNIP3 expression is regulating DR5 mRNA expression (Figure 2b).

### Identification of a BNIP3 binding region in the promoter region of DR5

**Figure 3:**
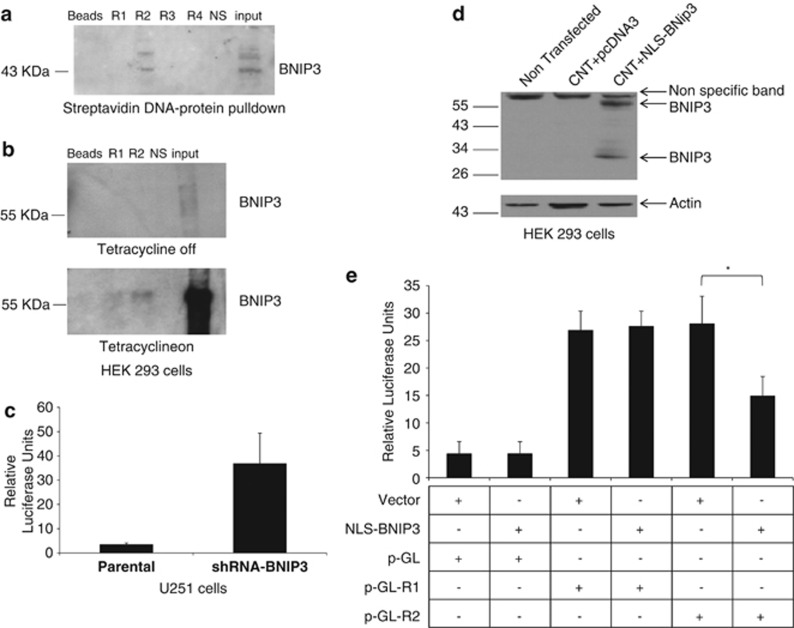
BNIP3 binds to a specific region in the DR5 promoter. (**a**) Oligonucleotide probes containing four different putative BNIP3 DNA binding sequences (Supplementary Table 1) from the promoter region of DR5 (R1-4), beads alone (beads) and a non-specific promoter region were incubated with recombinant BNIP3 protein. Input indicates lysate alone without beads or DNA. Interestingly, only the oligonucleotide containing region 2 of DR5 showed binding with BNIP3. (**b**) This pull-down experiment was repeated using lysates from HEK293 cells that stably express BNIP3 under the control of a Tet-ON promoter. Without tetracycline, BNIP3 expression is not seen in the beads alone, R1, R2 or the non-specific control. However, in the presence of tetracycline, binding is again observed in region 2. (**c**) A 60-bp fragment of the DR5 promoter was cloned into the luciferase reporter gene construct and then transfected into U251 parental cells, and U251 stably expressing shRNA against BNIP3. All cells were co-transfected with a beta-galactosidase vector for control of transfection efficiency. Luciferase activity was measured by a Softmax Pro Luminometer. These results are representative of three independent experiments. (**d**) HEK293 cells were transfected with BNIP3-NLS expression vector or vector alone (pcDNA3) in combination with luciferase vector containing the DR5 promoter region 1 or 2. Cells were lysed and western blotted for BNIP3 and actin as a loading control. (**e**) Transfected HEK293 cells were also measured for luciferase activity as described above. These results are representative of the three independent experiments. * represented statistical significance from three independent experiments

We have previously identified that BNIP3 can bind to DNA sequences identified from the ChIP DNA library.^29^ We found four regions within the DR5 promoter that could bind to nuclear BNIP3 (Supplementary Table 1). We chose the 18-bp sequence based on alignment of potential BNIP3 binding sites identified in the DR5 promoters, and these oligonucleotide probe was incubated with recombinant BNIP3 proteins. We observed a band with the wild-type BNIP3 protein corresponding with region 2 of the DR5 promoter (Figure 3a). To confirm these results, we used a tetracycline inducible system in HEK293 cells. The cells were lysed and incubated with oligonucleotide probes for the two regions on the DR5 promoter in the presence or absence of tetracycline. We found that BNIP3 bound to region 2 of the DR5 promoter after cells were induced by tetracycline (Figure 3b Supplementary Table 1). This indicates that nuclear BNIP3 binds to region 2 of the DR5 promoter.

To determine whether the nuclear BNIP3 is important for the regulation of DR5 gene transcription, a 60-bp fragment of the DR5 promoter was cloned into a luciferase reporter gene construct driven by a minimal CMV promoter to test whether this region can modulate expression of a reporter gene *in vitro*. This luciferase construct was transfected into U251 cell lines stably expressing vector alone, or shRNA against BNIP3 in a mammalian expression vector (lowers the levels of BNIP3 in the nucleus compared with parental). When BNIP3 expression was knocked down with shRNA, there was a significant increase in luciferase expression compared with controls (Figure 3c). As U251 cells express endogenous BNIP3, we used HEK293, cells that fail to express BNIP3, to overexpress BNIP3 with a NLS (NLS-BNIP3) together with a luciferase plasmids that contain regions 1 or 2 of the DR5 promoter (pGL-R1 or pGL-R2) (Figure 3d). This showed that NLS-BNIP3 significantly repressed luciferase activity in the DR5 promoter containing region 2 (Figure 3e). These data strongly implicate this region of the DR5 promoter in repression of DR5 gene expression.

### Nuclear BNIP3 expression blocks TRAIL-induced apoptosis

**Figure 4:**
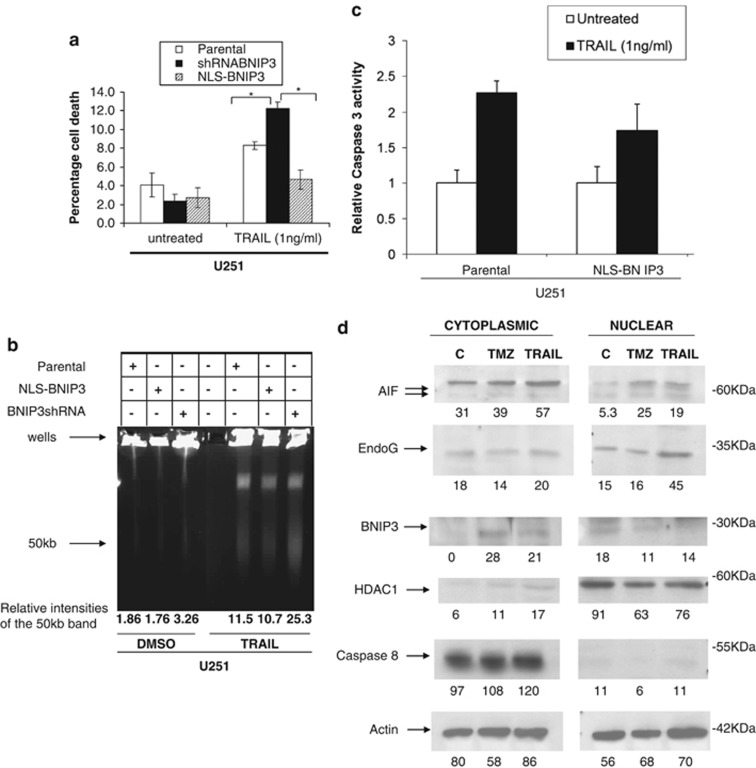
TRAIL-induced apoptosis is blocked by nuclear BNIP3. (**a**) U251 parental, U251shRNABNIP3 and U251NLS-BNIP3 stable cells were treated with 1 ng/ml of TRAIL for 24 h or DMSO for control. Percentage cell death was determined by acridine orange staining. Error bars represent the S.E. determined from three independent experiments. * denotes a *P*-value <0.05 representing statistical significance between U251 parental and U251shRNABNIP3, as well as between U251shRNABNIP3 and U251NLS-BNIP3. (**b**) U251 parental, U251shRNABNIP3 and U251NLS-BNIP3 stable cells were treated with 1 ng/ml of TRAIL for 24 h or DMSO for control, and then genomic DNA was extracted and run on a BioRad pulse field gel apparatus. The arrow indicates ∼ where the 50 kb DNA fragments migrate. This experiment was repeated three times and results were quantified by densitometry. (**c**) U251 parental and U251NLS-BNIP3 stable cells were treated with 1 ng/ml TRAIL for 24 h. Relative caspase 3 activity was measured as outlined in the Materials and Methods section. These results are representative of the three independent experiments. (**d**) Cytoplasmic and nuclear fractions were isolated from U251 cells that were untreated,or treated with TMZ and TRAIL as above. The lysates were western blotted for AIF, EndoG, BNIP3 (T7 antibody), HDAC1 (nuclear protein) and caspase 8 (cytoplasmic protein). Caspase 8 and HDAC1 were used for cytoplasmic and nuclear controls. The blots were stripped and reprobed with antibodies against actin for loading control

DR5 has an important role in TRAIL-induced apoptosis in glioma cells.^10^ To establish the functional consequences in glioma cells with altered DR5 levels due to nuclear BNIP3, we treated the U251 stable cell lines that have different nuclear BNIP3 levels with TRAIL (Figure 4). Cells with high nuclear BNIP3 were more resistant to TRAIL-induced cell death (Figure 4a). Correspondingly, cells with low nuclear BNIP3 expression were more sensitive to TRAIL-induced cell death (Figure 4a). To confirm these results, we used a pulsefield gel electrophoresis to detect 50 kb DNA fragmentation in the U251 stable cell lines. We discovered that cells that have high nuclear BNIP3 and low DR5 expression contain less DNA fragmentation than parental controls when treated with TRAIL. In cells that have low nuclear BNIP3 and high DR5 expression, increased DNA fragmentation was detected compared with parental controls (Figure 4b). To confirm that nuclear BNIP3-regulated DR5 expression leads to cell death, caspase 3 activity was measured in the U251 stable cell lines and parental controls after TRAIL treatment. There was activation of caspase 3 activity following TRAIL treatment, but caspase 3 activity was lower in the U251NLS-BNIP3 cell line (Figure 4c). We also tranfected cells with NLS-BNIP3 and shRNA against BNIP3. We found cleaved caspase 3 increased with TRAIL treatment and was further increased with the knockdown of BNIP3, whereas NLS-BNIP3 reduced cleaved caspase 3 (Supplementary Figure 3).

To determine whether mitochondrial release of proteins was affected by nuclear BNIP3 following TMZ or TRAIL treatment, TMZ and TRAIL-treated U251 cells were fractionated into nuclear and cytoplasmic lysates, and western blotted for AIF. We found that AIF protein expression in the nucleus increased after both TMZ and TRAIL treatment, indicating release from the mitochondria (Figure 4d). We also confirmed that NLS-BNIP3 was mainly expressed in the nuclear fraction (Supplementary Figure 5). An increase in AIF expression in the cytoplasm and in total cell extracts was also observed after both TMZ and TRAIL treatment (Figure 4d). Endonuclease G is also released from the mitochondria and localized in the nuclear following TRAIL treatment (Figure 4d). After treatment, BNIP3 levels in the nucleus decreased and increased in the cytoplasm (Figure 4d) similar to what happened under hypoxia. HDAC1 and caspase 8 antibodies were used as nuclear and cytoplasmic controls, respectively, to determine the amount of protein cross-contamination between nuclear and cytoplasmic lysates.

### Nuclear localized BNIP3 in primary human GBM tumors correlates with low DR5 expression

**Figure 5:**
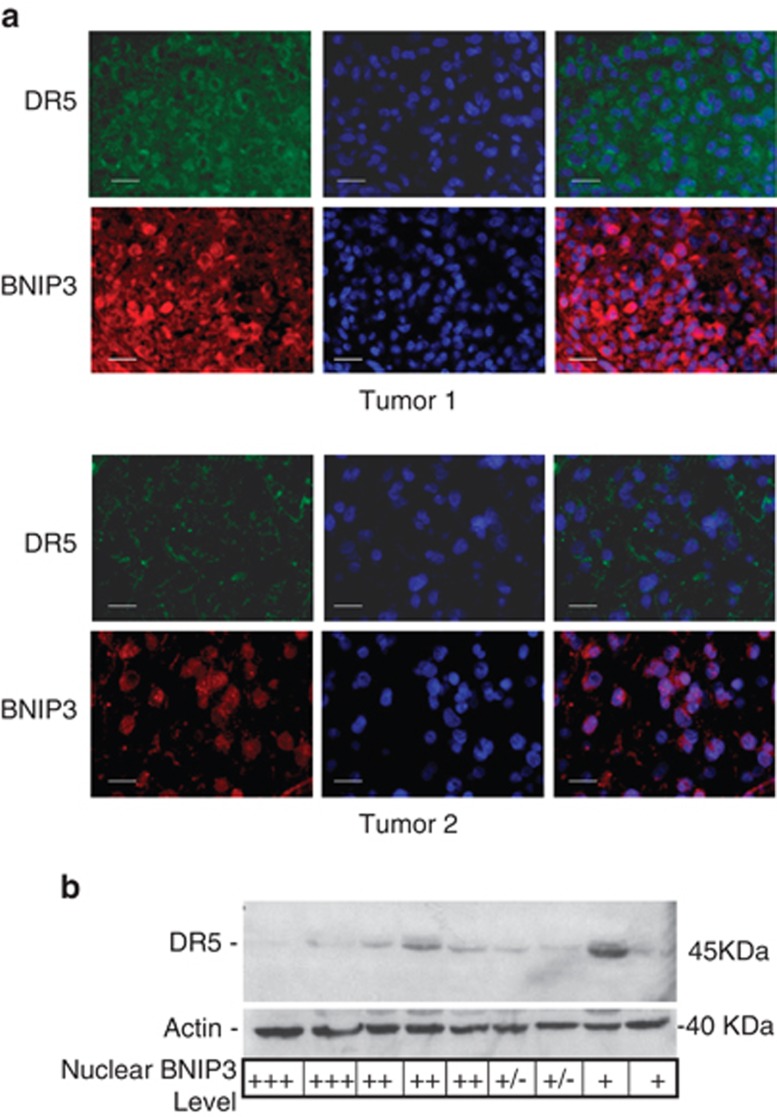
Primary GBM tumors that have high nuclear BNIP3 levels have low DR5 expression (**a**) Paraffin sections of representative primary GBM tumors sections were immunostained with antibodies against BNIP3 (red) and DR5 (green). DNA was stained with DAPI (blue), and the slides were analyzed on an Olympus fluorescence microscope. Scale bar represents 20 *μ*M. (**b**) Representative frozen GBM tumor tissues were lysed to extract total protein, and analyzed by western blot for DR5 expression. The blots were stripped and reprobed with actin antibodies for loading control. The grading for nuclear BNIP3 levels (determined by immunofluorescence) is indicated for each tumor. Nuclear staining was graded as: +++ for high nuclear staining, ++ for moderate nuclear staining,+for low nuclear staining, and +/− for undetectable nuclear staining. Three independent experiments were quantified by densitometry with Quantity One (Bio-Rad) and averaged to obtain DR5 expression relative to actin loading controls

Nuclear BNIP3 has been detected in GBMs,^28^ lung,^30^ and breast tumors.^31, 32^ DR5 expression has been observed to be altered in GBM tumors, and high expression for DR5 is associated with better survival in GBM patients.^33, 34^ As both nuclear localization of BNIP3 and DR5 expression showed variable patterns in GBM tumors, we asked whether nuclear BNIP3 correlated with levels of DR5 expression in GBM tumors. We immunostained formalin-fixed (FFPE) paraffin-embedded sections of primary GBM tumors with antibodies against BNIP3 and DR5. Tumors were counterstained for DNA with DAPI to identify the nucleus of the cells. To examine the localization of BNIP3 in GBM tumors, 14 tumors were graded according to whether BNIP3 was highly nuclear in localization, moderately nuclear or had low or no nuclear staining. The same tumors were graded for DR5 expression. Grading of the tumors was analyzed by a Chi-squared test and was statistically significant (*P*<0.05), supporting that tumors with high nuclear BNIP3 levels have low DR5 expression, whereas tumors that have low nuclear BNIP3 have correspondingly high DR5 expression (Table 1). Two representative GBM tumors containing high and low nuclear BNIP3 expression, respectively, were immunostained for DR5, showing that nuclear BNIP3 correlated with lower DR5 expression (Figure 5a). Consistent with these findings, nuclear BNIP3 expression was associated with lower DR5 expression by immunoblotting of lysates obtained from unfixed frozen GBM samples matched to the FFPE tumor sections (Figure 5b). Thus, these data provides strong evidence consistent with nuclear BNIP3 repression of the expression of the DR5 gene in GBM tumors.

## Discussion

We have discovered that normal astrocytes localize BNIP3 to the nucleus, preventing its pro-death function.^28^ This mechanism is exploited in GBM tumors, where upregulation of BNIP3 in the nucleus occurs in the majority of tumors (60%). Nuclear localization of BNIP3 is found in the lung,^30^ breast^31^ and cervical tumors,^35^ as well as in focal brain ischemia.^36^ In ductal carcinoma *in situ* of the breast, nuclear BNIP3 staining is present, but is significantly reduced in invasive breast tumors.^31^ In addition, nuclear BNIP3 was significantly correlated with a shorter disease-free survival.^31^ It was observed that nuclear localization of BNIP3 occurred in a subset of cases that had a particularly poor prognosis.^30^ These translational research studies provide strong evidence that nuclear localized BNIP3 in tumor cells is a phenotype selected to enhance the survival of tumor cells. We have now discovered that BNIP3 has a role in repressing DR5 expression and blocking TRAIL-induced apoptosis.

Cancer-specific molecules have been identified and used as potential targets for GBM therapy. A particularly promising novel therapeutic approach for GBM is the activation of the death receptor pathway through the treatment with the death receptor ligand TRAIL. TRAIL is an effector molecule involved in immune surveillance and is important for the elimination of virally infected and cancer cells.^6, 7, 8^ The ability of TRAIL to induce apoptosis in normal cells appears very limited, where it has been shown to induce apoptosis in glioma cells. Recombinant versions of TRAIL have advanced into clinical trials for a variety of solid tumors. GBM is an attractive target for TRAIL therapy owing to the expression of DR5 and to a lesser extent DR4. The expression levels of these receptors have also been correlated with longer survival times for GBM patients.^9^ Unfortunately, many glioma cells are resistant to TRAIL-induced apoptosis, putting into question the clinical usefulness of TRAIL as a treatment. Our discovery that nuclear BNIP3 represses DR5 expression in both glioma cells and normal astrocytes, suggests that TRAIL treatment could be effective if nuclear BNIP3 transcriptional repression was inhibited.

Novel mechanisms for Bcl-2 family members in the nucleus have been described. The BH3-only member of the Bcl-2 family BID is localized to the nucleus and has a role in the DNA damage response, and regulates the cell cycle.^37^ In addition, nuclear Bcl-2 inhibits transcription factor activation and alters the expression of DNA repair enzymes.^38, 39^ We have previously identified that nuclear BNIP3 acts as a transcriptional repressor binding to the AIF promoter, thereby preventing apoptosis. Indeed, the region where BNIP3 binds contains a sequence that is homologous to a consensus repressor signal for neural-specific genes.^40^ We have found similar regions within the DR5 promoter and in cells knocked down for nuclear BNIP3, the promoter activity was increased. Besides AIF and DR5, BNIP3 may bind to multiple promoters and alter gene expression in many different types of cancer cells. Indeed, AIF expression is affected by reduced BNIP3 expression mediated by microRNA 145 in prostate cancer cells.^41^ Nevertheless, this unique BNIP3 repressor function for DR5 gene alters TRAIL-induced apoptosis in glioma cells and could be an important mechanism for TRAIL resistance in GBM tumors.

Besides transcription factors upregulating DR5 expression, transcriptional repressors have been implicated in regulating DR5 expression, but are less well characterized. The transcriptional repressor Yin Yang 1 binds to the DR5 promoter and blocks DR5 transcriptional activation.^42^ In addition, HDAC blocks gene transcription by deacetylating both histones and transcription factors.^43^ Under growth factor stimulation, HDAC1 is recruited to the DR5 gene, whereas under apoptotic stimuli HDAC1 is not recruited.^44^ This differential recruitment is mediated by NF*κ*B, where under growth factor stimulation NF*κ*B binds to HDAC1 and the DR5 gene, whereas NF*κ*B fails to bind to HDAC1 under apoptotic conditions. BNIP3 also represses DR5 expression and we have previously shown that BNIP3 forms a complex with HDAC1. HDAC inhibitors such as VPA are effective at inducing cell death in glioma cells and increase AIF expression, suggesting inactivation of the BNIP3 repressor activity.^29^ Furthermore, DR5 expression is increased by HDAC inhibitors.^44^ These studies indicate that DR5 expression is dynamically regulated and BNIP3 has a role in controlling DR5 expression in glioma cells and astrocytes.

Overall, nuclear BNIP3 downregulates DR5 expression in glioma cells, leading to resistance to TRAIL-induced cell death. This may be relevant to GBM tumor cell survival because BNIP3 is primarily localized in the nucleus in the majority of GBM tumors and correlates with lower levels of DR5 expression. This provides a novel and potentially important mechanism for repressing DR5 expression and could be a future target for therapy rendering GBM tumor sensitivity to TRAIL therapy.

## Materials and Methods

### Cell culture and transfections

Human glioblastoma cell lines U251 (obtained from Dr. VW Yong, University of Calgary and Dr. C Hao, Emory University, respectively) were cultured in Dulbecco's modified essential medium (DMEM), supplemented with 10% fetal bovine serum, 2 mM ℒ-glutamine, 1 mM MEM sodium pyruvate, 0.3% glucose and 100 units/ml penicillin/streptomycin. Mouse astrocytes were passaged in DMEM/F12 supplemented with 10% fetal bovine serum and 100 units/ml penicillin/streptomycin. The cell lines were grown in a humidified incubator in the presence of 5% CO_2_ at 37 °C. In transfection experiments, the cells were plated 48 h before transfection to achieve ∼60% confluence. The HEK293 cell line was transfected with Lipofectamine (Invitrogen; Life Sciences, Burlington, ON, Canada), the U87 cell line was transfected using Gene porter (GTS) and the U251 cell line using Effectene (Qiagen, Toronto, ON, Canada) as per the manufacturer's instructions. Cells were plated 48 h before transfection in six-well dishes so that they were between 50 and 70% confluent before transfection. Two micrograms of DNA was diluted in DNA Diluent B, mixed by pipetting and incubated at room temperature for 5 min and then added to the gene porter 2 reagents and incubated for 10 min before adding to the cells. Stable cell lines were derived in U251 cells by transfecting with pSUPER plasmid containing shRNA for BNIP3 or non-targeting shRNA control and pCDNA3 containing NLS tagged BNIP3 or vector alone control, and selecting with 1.5 mg/ml G418 (Gibco BRL; Life Sciences). GFP containing vector was transient transfections were done using Gene Porter 2 (Genlantis, Interscience, San Diego, ON, USA) as per manufactures instructions.

### Cryopreservation of mouse brains

Mice were perfused with paraformaldehyde and the brains removed and fixed overnight in 4% paraformaldehyde and then the brains were paraffin embedded, and slides prepared by the Manitoba Tumor Bank.

### Isolation of astrocytes

Newborn mice were killed by decapitation and then the brains were removed and placed in ice-cold serum-free media. The cortex was then dissected out and chopped into small pieces and then vortexed and filtered through 70 and 10 *μ*m filters in sequence. Cells were then diluted in media with serum at a final concentration of 2–5 × 10^5^/ml. Cultures were kept at 37 °C, 5% CO_2_ changing the media after the first 3 days, and then twice a week after that. During media changes, the dishes were swirled to detach contaminating glial cells from the astrocytes. Cultures became confluent between 10 and 14 days and reached functional maturity 14 days after that. Cells were stained with GFAP to confirm the purity of culture.

### Plasmids

A partial DR5 promoter sequence was inserted into the pGL3 promoter vector and pGL3 control vector, as well as a scrambled control using Nhe1 and Xho1 restriction enzymes.

DR5 promoter region: Fwd:5′-CTAGCCTCTCCCGCGGCGGGCCTTCCCCATTGGCCAGCCAAACACAACCGACTGCGGCAGCCAC-3′

Rev:5′-TCGAGTGGCTGCCGCAGTCGGTTGTGTTTGGCTGGCCAATGGGGAAGGCCCGCCGCGGGAGAGG-3′

The mutations in the NLS-BNIP3 plasmid were created by QuikChange site-directed mutagenesis (Stratagene; Agilent, Santa Clara, CA, USA ), deleting 5 amino acids (IERRK, 100–104) with primers:

Fwd:5′-GCTCACAGTCTGAGTCTGAGGAAGATGATGAAGTTGAAAGCATC-3′

Rev:5′-GATGCTTTCAACTTCATCATCTTCCTCAGACTCAGACTGTGAGC-3′.

PCR: 18 cycles, 95 °C 30 s, 55 °C 1 min, 68 °C 5.6 min. A His-tag was added into the wild-type pCDNA3 BNIP3 construct with primers:

Fwd: 5′-ggagcgcccgggCATCATCACCATCACCATatgcaggaggag-3′

Rev: 5′- ctcctcctgcatATGGTGATGGTGATGATGcccgggcgctcc-3′

with the same site-directed mutagenesis protocol as the NLS-BNIP3 plasmid.

A 19-bp section of the BNIP3 sequence was constructed into a short hairpin RNA and cloned into the pSUPER vector with BglII and HinDIII restriction sites, to obtain an expression vector for BNIP3shRNA in mammalian cells.

#### Sequence

5′-gatccccGCCTCGGTTTCTATTTATAttcaagagaTATAAATAGAAACCGAGGCttttta-3′

### Western blotting

Cell lines (U251, U87 and HEK293), primary mouse astrocytes and frozen primary GBM tissue samples Brain Tumor Tissue Bank (London, ON, Canada) (BTTB) were lysed for total proteins, membrane proteins or nuclear proteins as previously published.^28^ The lysates (60 *μ*g) were separated by SDS-PAGE and transferred to nitrocellulose membranes. Membranes were blocked in 5% skim milk and western blotted with monoclonal antibodies against BNIP3 (1 : 1000, preferentially recognizes the 30 kDa protein) (5), T7 tag (1 : 2000, MBL), DR5 (1 : 1000, Upstate Biotech; Millipore, Billerica, MA, USA), BID (1 : 1000, Cell Signaling), TRAIL (1 : 200, Santa Cruz, Dallas, TX, USA), DR4 (1 : 200, Santa Cruz), Caspase 3 (1 : 1000, Cell Signaling, Danvers, MA, USA), Caspase 8 (Santa Cruz, 1 : 200), FLIP (1 : 1000, Abcam, Toronto, ON, Canada), Bcl-xL (1 : 1000, Cell Signaling), FAS(1 : 1000, Cell Signaling), HDAC1 (1 : 1000, Millipore), AIF (1 : 1000, Cell Signaling), Endonuclease G (1 : 1000, Cell Signaling), His tag (1 : 200 Santa Cruz), or Actin (1 : 50, Sigma, Oakville, ON, Canada). The western blots were visualized with chemiluminescence (NEN-Dupont, Boston, MA, USA).

### Real time RT-PCR

RNA was isolated from U251, U251shRNABNIP3 and U251NLS-BNIP3 cells with 1 ml RNA-Bee (TEL-TEST, Inc. Friendswood, TX, USA) per sample. Chloroform (200 *μ*l) was used to isolate the DNA and isopropanol (0.5 ml) to precipitate the RNA. The pellet was then resuspended in sterile ddH_2_O and stored at −80 °C. Real time PCR was performed with a iCycler using the Biorad iScript SYBR green kit to measure DR5 and cyclophilin mRNA levels in U251, U251shRNABNIP3 and U251NLS-BNIP3 stable cells. RNA was isolated from the brain samples from mice stored in RNA later by spinning down and removing the RNA later from the tissue and then isolating RNA using the RNA-Bee protocol described above. Real time PCR was performed as described above to measure DR5 and 18s mRNA levels in wild type and BNIP3 knockout mouse brains.

#### Sequences for real time PCR

GAPDH: 5′-AGGTCGGTGTGAACGGATTTG-3′ and 5′-TGTAGACCATGTAGTGGTCA-3′

18S: 5′-TACCACATCCAAGAAGGCAG-3′ and 5′-TGCCCTCCAATGGATCCTA-3′

DR5: 5′-GTCAGAAGGGAACTGCAAGC-3′ and 5′-GCATCGACACACCGTATTTG-3′

BNIP3: 5′-GCTCCCAGACACCACAAGAT-3′ and 5′-TGAGAGTAGCTGTGCGCTTC-3′

### His-tag pull-downs, streptavidin DNA-protein pull-downs and immunoprecipitation

Ni-NTA agarose beads (Qiagen) were resuspended in 1 ml wash buffer (10 mM Tris pH8, 100 mM NaCl, 0.1% NP40 5 mM Imidazole). His-tagged BNIP3 was bound to the Ni beads by incubation of 100 *μ*g of protein with the beads at 4 °C for 2 h with gentle shaking. The mixture was then washed three times with wash buffer and 500 *μ*g of U251 cell lysate was added to the beads to a total volume of 500 *μ*l, and incubated o/n at 4 °C. After incubation beads were washed 3 × with wash buffer and then proteins that were bound to the beads were eluted with 1 ml of 250, 500 and 800 mM imidazole, incubated for 10 min at 4 °C. Fractions were western blotted for the detection of proteins described above.

Streptavadin DNA-protein pull-downs were done as previously described.^45^ Briefly, biotin-labeled oligonucleotides were ordered from IDT technology. Oligos were resuspended in TE buffer and diluted to a final volume of 1 *μ*M and then annealed by heating to 95 °C for 5 min, 65 °C for 30 min and then room temperature (RT). Recombinant BNIP3 protein was then precleared with streptavidin-agarose beads for 30 min at 4 °C, and then incubated with the appropriate oligonucleotide mixture in HGE buffer (100 mM HEPES, 50% glycerol, 10 mM EDTA and 250 mM KCl) supplemented with 1%NP40, 0.5 mg/ml BSA, 50 ng/*μ*l poly dIdC, and 5 *μ*M DTT. The binding reaction was then incubated at room temperature for 30 min with rotation and then streptavidin-agarose beads were added and the reaction was incubated for 30 min at room temperature. Beads were spun down and washed three times with ice-cold PBS and then resuspended in 50 *μ*l of 2 × loading dye boiled, and then western blotted as previously described.^45^

For immunoprecipitations, U251 cell lysate (500 *μ*g) was precleared with 50 *μ*l rabbit TrueBlot IP beads (eBioscience, San Diego, CA, USA). The lysate was then immunoprecipitated with 5 *μ*g anti-HDAC1 antibody, or 5 *μ*g of PSF antibody, incubated o/n at 4 °C. Rabbit TrueBlot IP beads were added and incubated at 4 °C for 2 h. Beads were washed three times with lysis buffer to remove contaminants, and prepared for western analysis by adding Laemmli buffer with 2% beta-mercaptoethanol to the bead pellet and heating at 100 °C for 10 min.

### Cell death assays

#### Acridine orange staining

Cells were trypsinized, removed from the culture plates and centrifuged in 15 ml sterile tubes. The cells were then resuspended in 100 *μ*l of media by gently vortexing, and 4 *μ*l of acridine orange (100 *μ*g/ml) and ethidium bromide (100 *μ*g/ml) were added. A 10 *μ*l aliquot was removed and placed on a microscope slide, and a coverslip was applied. The slide was viewed on a fluorescence microscope using a fluorescein filter. The percentage of dead cells was calculated by counting the number of orange stained cells and cells containing bright green local DNA condensation in a population of diffuse green cells. At least 200 cells were counted for each experiment.^24^

### Immunofluorescence

FFPE primary GBM tumor section slides (BTTB) were baked in an oven (70 °C) for 20 min. The slides were deparaffinized, rehydrated and washed with H_2_O for 5 min. Antigen presentation was completed by incubating the slides in a pressure cooker for 20 min filled with citrate buffer (10 mM citric acid monohydrate, pH to 6.0). The slides were removed, cooled to RT, and then washed three times for 5 min in PBS-T (0.5% Triton X100). Blocking solution (1X PBS, 0.2% Triton X100, 0.02% sodium azide, 5% goat serum and 0.1% bovine serum albumin) was added to each slide for 2 h at RT. Primary antibodies (polyclonal anti-BNIP3 1 : 700 dilution, anti-DR5 1 : 1000 dilution (Cell Signaling) were diluted in blocking solution and added to slides. The slides were incubated at 4 °C overnight and subsequently washed. The appropriate biotinylated secondary antibody (1 : 200 dilution, Vector Labs, Burlington, ON, Canada) was prepared in blocking solution and added to the slides for 2 h at RT then washed three times. Stretavidin conjugated to the appropriate fluorochrome (Texas red or Oregon green, Burlington, ON, Canada) in blocking solution (15 *μ*g/ml) was added to slides and incubated for 2 h at RT in the dark. Vectashield with DAPI stain (Vector) was added to each slide and a coverslip was placed over the tumor sections and sealed. Fluorescence was visualized and captured using an Olympus BX51 fluorescent microscope with a Photometrics Cool Snap CF camera.

### Immunohistochemistry

Whole-brain sections from wild-type and BNIP3 knockout mice were prepared by the Manitoba Tumor Bank. Slides were baked in an oven (70 °C) for 20 min. The slides were deparaffinized, rehydrated and washed with H_2_O for 5 min. Antigen presentation was completed by incubating the slides in a pressure cooker for 20 min filled with citrate buffer (10 mM citric acid monohydrate, pH to 6.0). Slides were stained using the Dako EnVision FLEX system (Burlington, ON, Canada) as per the manufacture's instructions. DR5 antibody (Atlas) was diluted at 1 : 150.

### Luciferase assays

HEK293 cells, U251, U251shRNABNIP3 and U251NLS-BNIP3 stable cells were transiently transfected with the pGL3 promoter luciferase reporter vector and the pGL3 promoter vector with the DR5 promoter region 1 or 2 that contains the BNIP3 binding sites identified by ChIP. All cells were co-transfected with a beta-galactosidase vector measured at 414 nm for the control of transfection efficiency. Cells were lysed with cell culture lysis reagent (Promega, Madison, WI, Canada) for 15 min at RT. Luciferase activity was measured by a Softmax Pro Luminometer (Molecular Devices Corporation, Sunnyvale, CA, USA) for 10 s of relative light units. Results were normalized relative to beta-gal activity. *T*-tests were performed to determine significance.^44^

### Pulsefield gel electrophoresis

U251, U251shRNABNIP3 and U251NLS-BNIP3 stable cells were treated with DMSO for control, and 1 ng/ml TRAIL (Axxora) for 24 h. Genomic DNA was extracted using QIAamp DNA mini kit (Qiagen) and run on a 1% megabase agarose (Bio-Rad, Mississauga, ON, Canada) gel using the BioRad CHEF-DR II pulse field gel apparatus. The chamber was filled with 0.5% TBE buffer that was maintained at temperatures below 14 °C with tubing coiled in an ice bath. The run parameters were set at 250 V with 10 s pulse for 30 h. When the run was completed the gels were stained in a 0.5 *μ*g/ml ethidium bromide solution for 30 min. and destained in ddH_2_O for 3 h. DNA was visualized by placing the gel on a UV transilluminator (254−360 nm). Results were quantified by densitometry.

### Caspase 3 activity assay

Caspase 3 activity was measured using the Caspase 3 assay kit from Abcam (ab39383) as per manufactures instructions. Briefly, samples were trypsinized and resuspended in cell lysis buffer and allowed to lyse for 10 min. Reaction buffer containing DTT and the DEVD-AFC substrate was added and the mixture was incubated at 37 °C for 2 h and then the samples were read on a SpectraMax GeminiXS using a 400 nm excitation and 505 nm emission filter.

## Figures and Tables

**Table 1:** DR5 expression levels in GBM tumors correlates with nuclear BNIP3 levels

	*DR5 expression*			
*BNIP3 localization*	*Expressed (0–1*+)	*Overexpressed (2*+)	*Strongly overexpressed (3*+)	*Total*
*Actual*				
Nuclear (3+)	3	3	1	7
Moderate nuclear (2+)	9	0	0	9
Low nuclear (0–1+)	6	2	6	14
				
*Expected*				
Nuclear (3+)	2.33	2.33	2.33	7
Moderate nuclear (2+)	3	3	3	9
Low nuclear (0–1+)	4.67	4.67	4.67	14
			Chi-squared	0.00026

A total of 14 GBM tumors were scored for BNIP3 localization and then graded for DR5 expression levels. We predict that there would be an even distribution of DR5 expression across the different levels of BNIP3; however, tumors that had high levels of BNIP3 in the nucleus had lower levels of DR5, and when there were lower levels of BNIP3 in the nucleus there were higher levels of DR5. Chi square analysis showed that these differences were highly significant (*P*<0.001).

## Abbreviations

AIF-1: apoptosis-inducing factor
BNIP3: Bcl-2 nineteen kilodalton interacting protein 3
BTTB: Brain Tumor Tissue Bank
DCIS: ductal carcinoma *in situ*
DMEM: Dulbecco's modified essential medium
DR4: death receptor-4
DR5: death receptor-5
FFPE: formalin-fixed paraffin-embedded
GBM: glioblastoma multiforme
HDAC: histone deacetylase
HDI: histone deacetylase inhibitors
Δψm: mitochondrial membrane potential
miR145: microRNA 145
NS: non-specific promoter region
PCD: programmed cell death
PT: permeability transition
ROS: reactive oxygen species
TRAIL: Tumor necrosis-related apoptosis-inducing ligand
TMZ: temozolomide
Wt: wild type
YY1: Yin Yang 1
