# Supplementary material for: BNIP3 acts as transcriptional repressor of death receptor-5 expression and prevents TRAIL-induced cell death in gliomas
Source: Cell Death Dis. 2013 Apr 11;4(4):e587–. doi: 10.1038/cddis.2013.100 (PMC3641324; doi:10.1038/cddis.2013.100)
Supplement: Supplementary Figures and Table [file cddis2013100x1.ppt]

## Slide 1
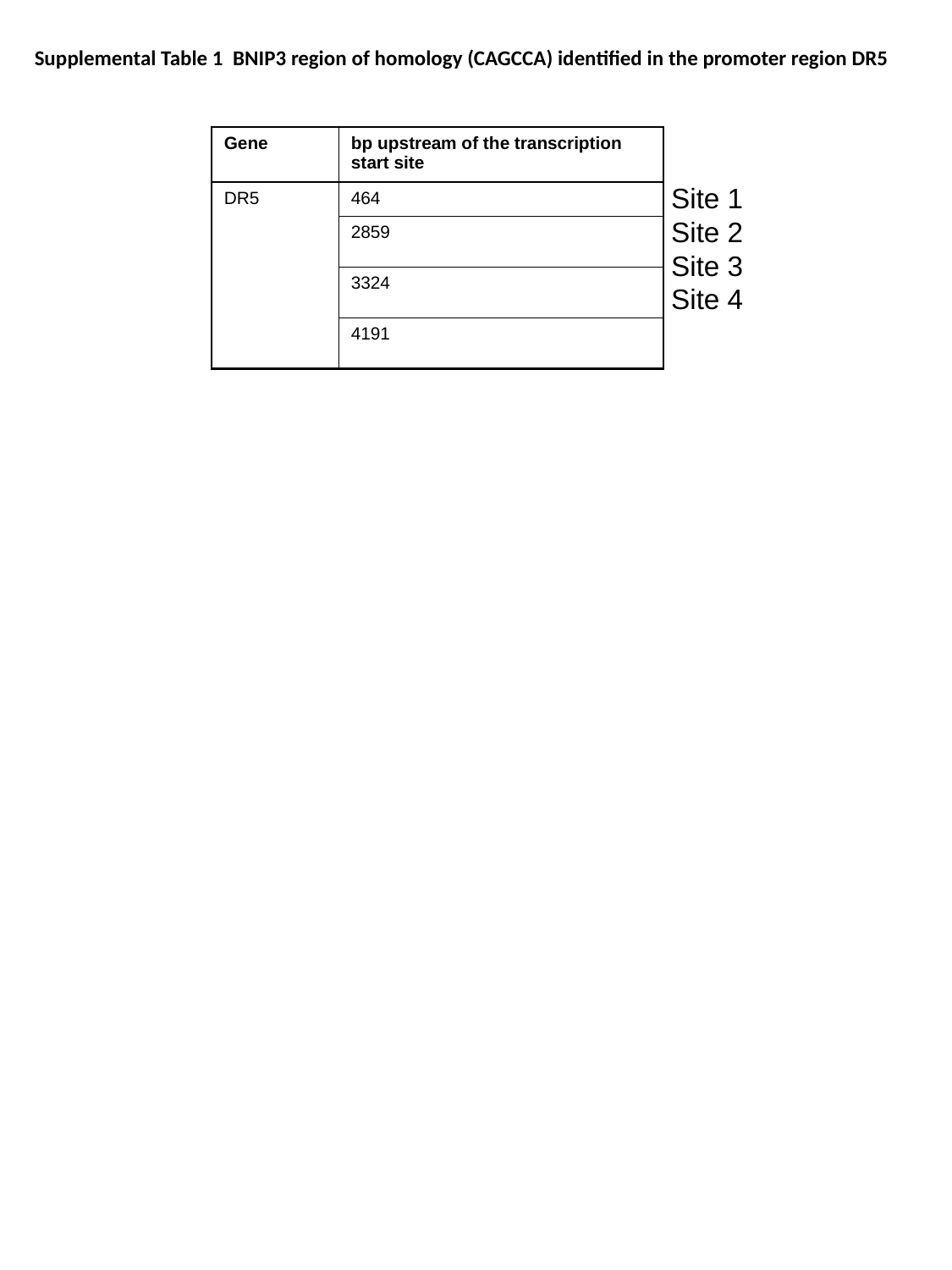

Supplemental Table 1 BNIP3 region of homology (CAGCCA) identified in the promoter region DR5
| Gene | bp upstream of the transcription start site |
| --- | --- |
| DR5 | 464 |
| | 2859 |
| | 3324 |
| | 4191 |
| | |
| | |
| | |
| | |
| | |
| | |
| | |
| | |
| | |
Site 1
Site 2
Site 3
Site 4

## Slide 2
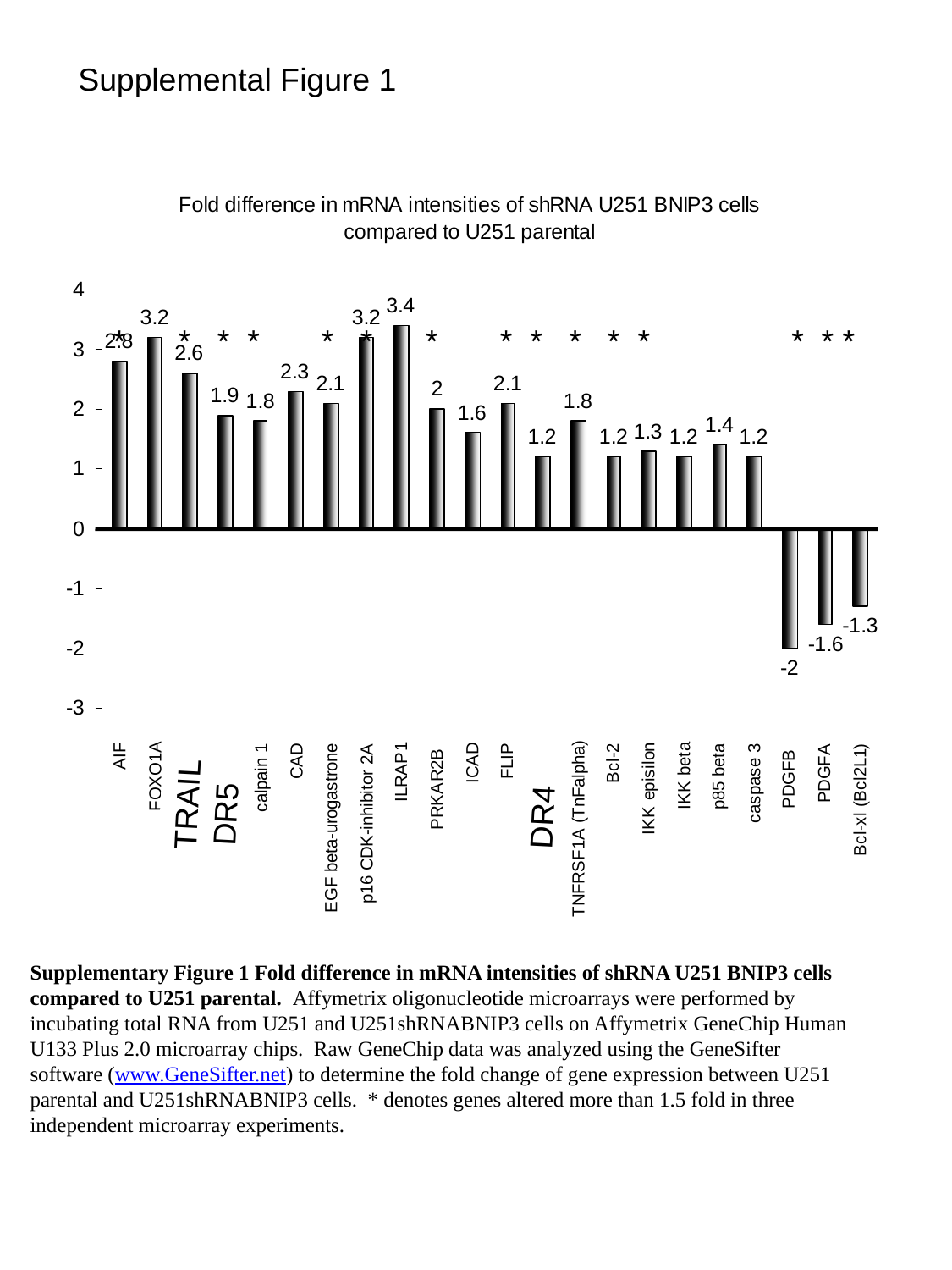

Supplemental Figure 1
* * * * * * * * * * * * * * *
DR5
 TRAIL
 DR4
Supplementary Figure 1 Fold difference in mRNA intensities of shRNA U251 BNIP3 cells compared to U251 parental. Affymetrix oligonucleotide microarrays were performed by incubating total RNA from U251 and U251shRNABNIP3 cells on Affymetrix GeneChip Human U133 Plus 2.0 microarray chips. Raw GeneChip data was analyzed using the GeneSifter software (www.GeneSifter.net) to determine the fold change of gene expression between U251 parental and U251shRNABNIP3 cells. * denotes genes altered more than 1.5 fold in three independent microarray experiments.

## Slide 3
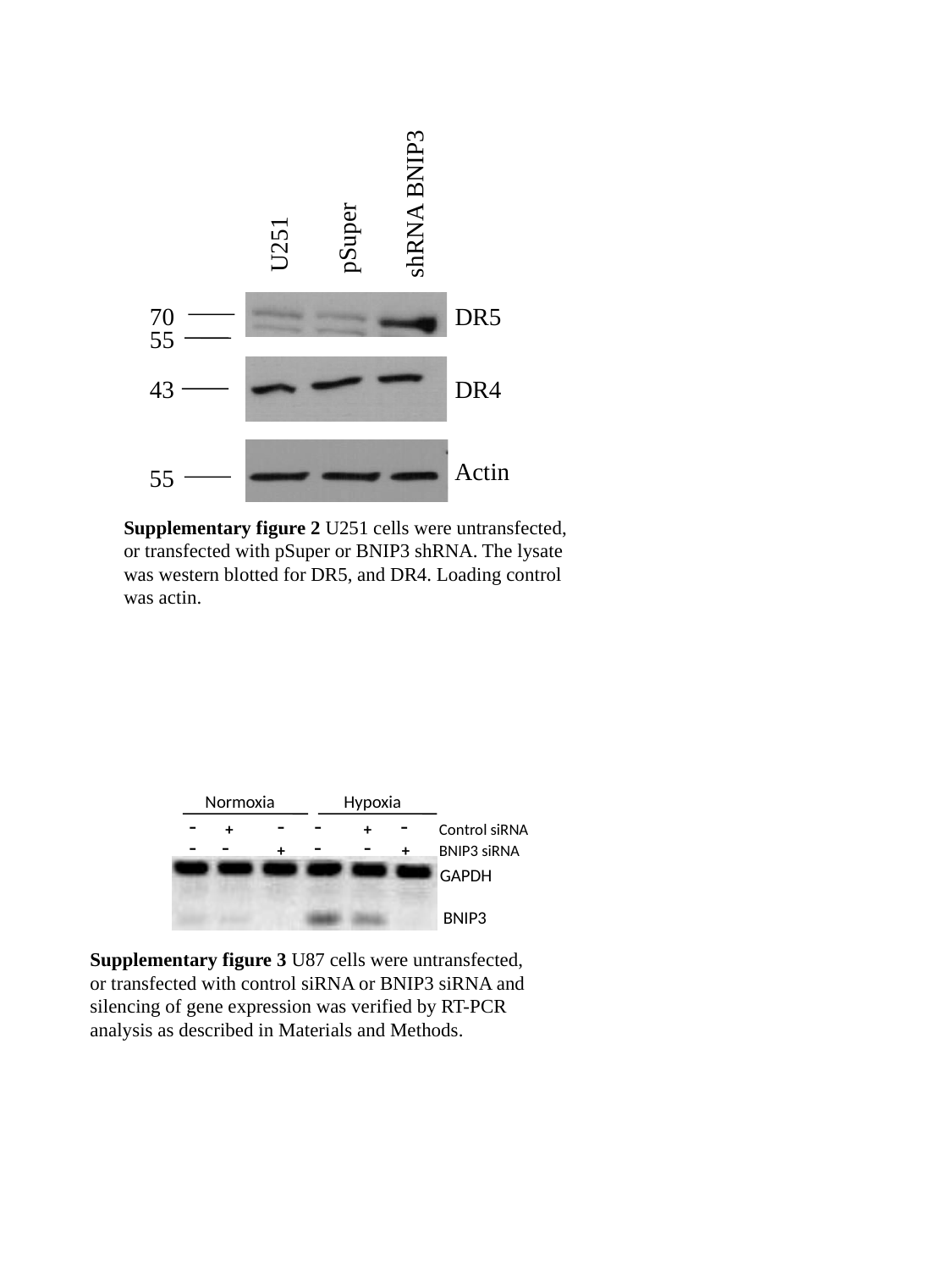

shRNA BNIP3
pSuper
U251
70
DR5
55
DR4
43
Actin
55
Supplementary figure 2 U251 cells were untransfected, or transfected with pSuper or BNIP3 shRNA. The lysate was western blotted for DR5, and DR4. Loading control was actin.
Normoxia
Hypoxia
 	- + 	- 	- 	+ -	Control siRNA
 	- - 	+ - 	- 	+	BNIP3 siRNA
GAPDH
BNIP3
Supplementary figure 3 U87 cells were untransfected, or transfected with control siRNA or BNIP3 siRNA and silencing of gene expression was verified by RT-PCR analysis as described in Materials and Methods.

## Slide 4
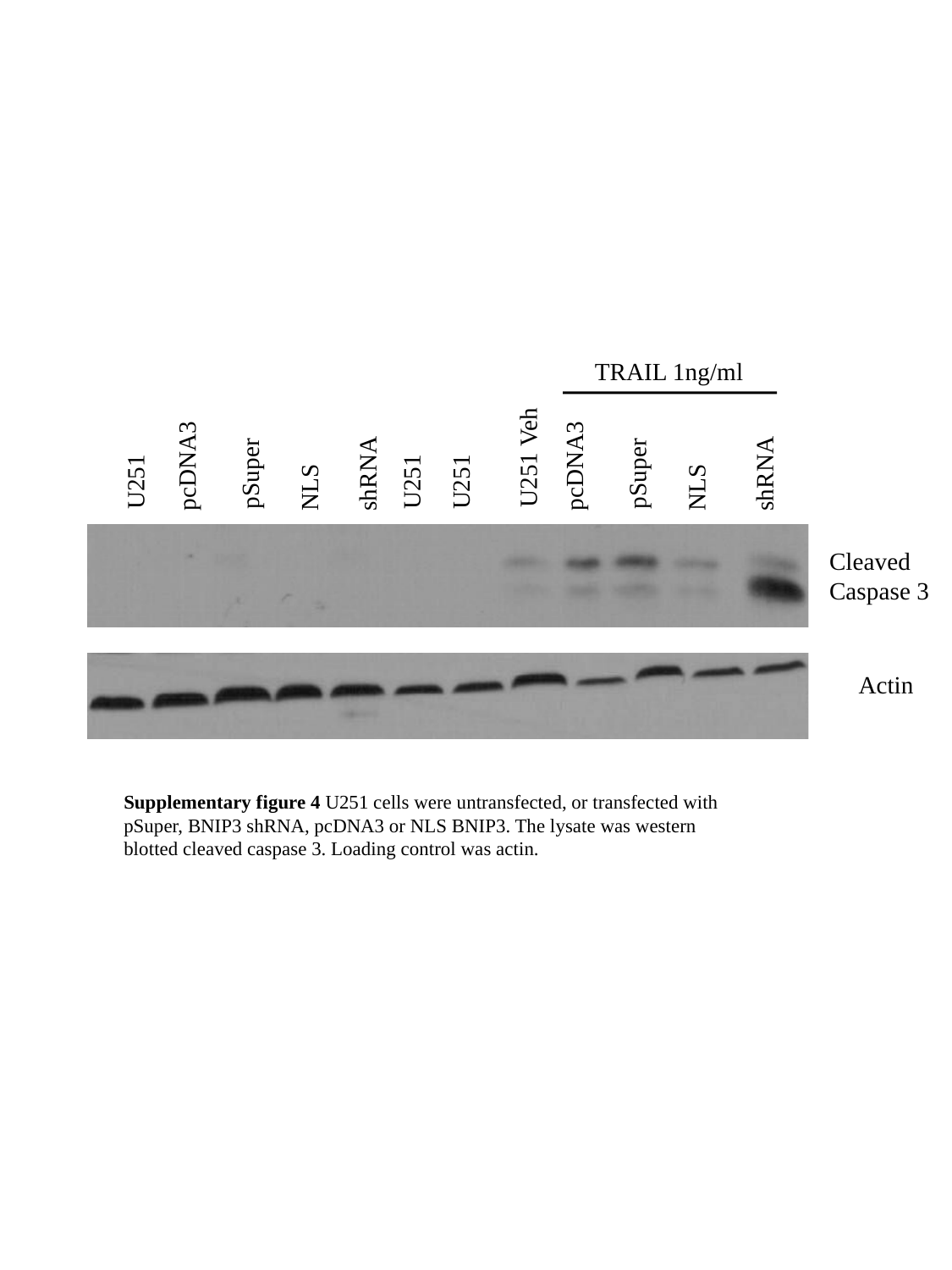

TRAIL 1ng/ml
U251 Veh
pcDNA3
pcDNA3
pSuper
shRNA
pSuper
shRNA
U251
U251
U251
NLS
NLS
Cleaved
Caspase 3
Actin
Supplementary figure 4 U251 cells were untransfected, or transfected with pSuper, BNIP3 shRNA, pcDNA3 or NLS BNIP3. The lysate was western blotted cleaved caspase 3. Loading control was actin.

## Slide 5
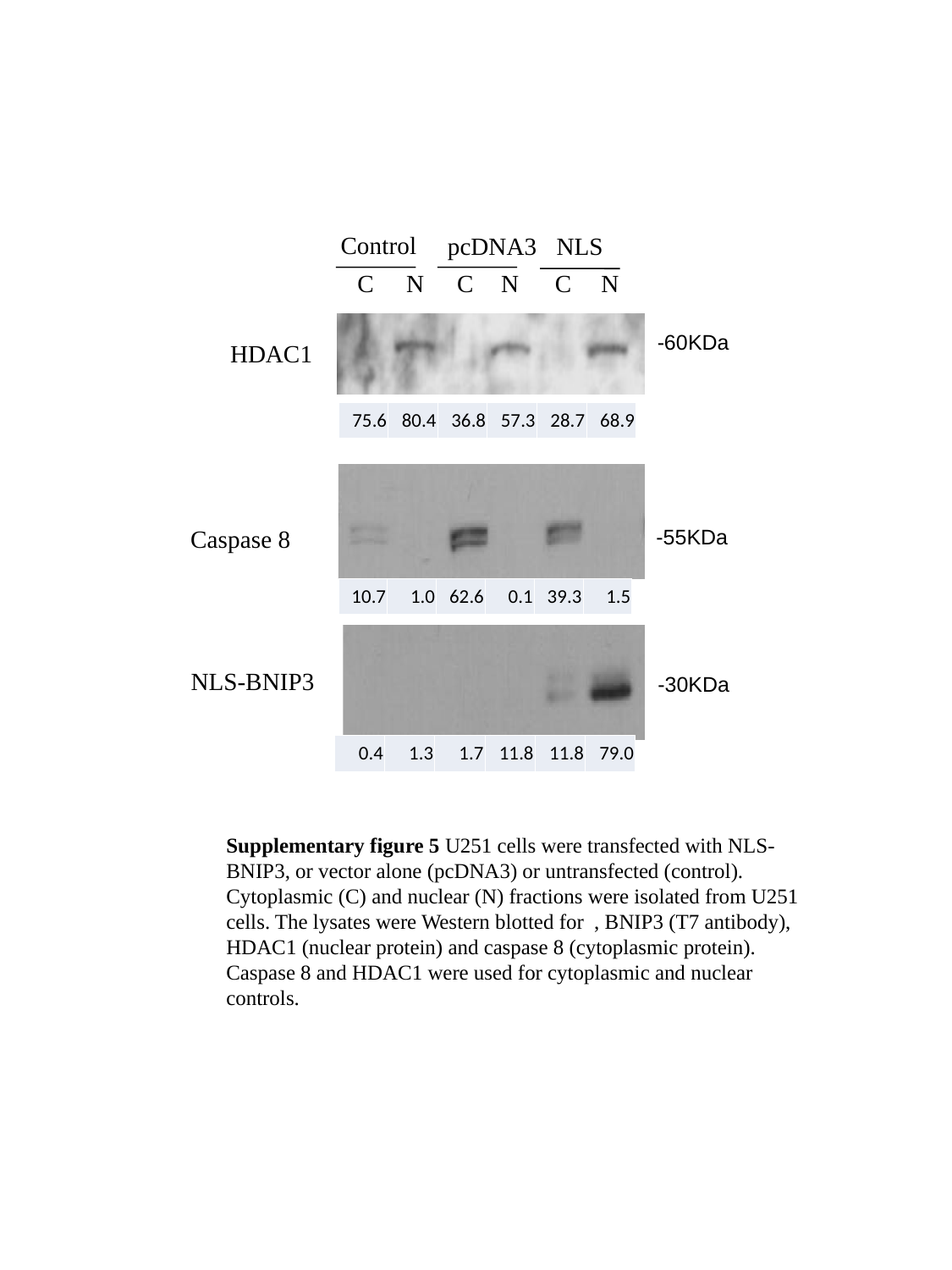

Control
pcDNA3
NLS
C
N
C
N
C
N
-60KDa
HDAC1
| 75.6 | 80.4 | 36.8 | 57.3 | 28.7 | 68.9 |
| --- | --- | --- | --- | --- | --- |
Caspase 8
-55KDa
| 10.7 | 1.0 | 62.6 | 0.1 | 39.3 | 1.5 |
| --- | --- | --- | --- | --- | --- |
NLS-BNIP3
-30KDa
| 0.4 | 1.3 | 1.7 | 11.8 | 11.8 | 79.0 |
| --- | --- | --- | --- | --- | --- |
Supplementary figure 5 U251 cells were transfected with NLS-BNIP3, or vector alone (pcDNA3) or untransfected (control). Cytoplasmic (C) and nuclear (N) fractions were isolated from U251 cells. The lysates were Western blotted for , BNIP3 (T7 antibody), HDAC1 (nuclear protein) and caspase 8 (cytoplasmic protein). Caspase 8 and HDAC1 were used for cytoplasmic and nuclear controls.
